# The correlation between serum selenium, zinc, and COVID-19 severity: an observational study

**DOI:** 10.1186/s12879-021-06617-3

**Published:** 2021-09-03

**Authors:** Soodeh Razeghi Jahromi, Hedieh Moradi Tabriz, Mansoureh Togha, Shadi Ariyanfar, Zeinab Ghorbani, Sima Naeeni, Samaneh Haghighi, Aboozar Jazayeri, Mahnaz Montazeri, Mohammad Talebpour, Haleh Ashraf, Mehdi Ebrahimi, Azita Hekmatdoost, Elham Jafari

**Affiliations:** 1 Department of Clinical Nutrition and Dietetics, Faculty of Nutrition and Food Technology, Shahid Beheshti University of Medical Sciences, Tehran, Iran; 2 Present Address: Department of Pathology, Sina Hospital, Tehran University of Medical Sciences, Tehran, Iran; 3 Headache Department, Iranian Center of Neurological Research, Neuroscience Institute, Tehran University of Medical Sciences, Tehran, Iran; 4 Neurology ward, Sina Hospital, Tehran University of Medical Sciences, Tehran, Iran; 5 Cardiovascular Diseases Research Center, Department of Cardiology, Heshmat Hospital, School of Medicine, Guilan University of Medical Sciences, Rasht, Iran; 6 Department of Clinical Nutrition, School of Medicine, Guilan University of Medical Sciences, Rasht, Iran; 7 Department of Infectious Diseases, Sina Hospital, Tehran University of Medical Sciences, Tehran, Iran; 8 Department of Surgery, Sina Hospital, Tehran University of Medical Sciences, Tehran, Iran; 9 Research Development Center, Sina Hospital, Tehran University of Medical Sciences, Tehran, Iran; 10 Cardiac Primary Prevention Research Center (CPPRC), Cardiovascular Diseases Research Institute, Tehran University of Medical Sciences, Tehran, Iran; 11 Endocrinology Department, Sina Hospital, Tehran University of Medical Sciences, Tehran, Iran

**Keywords:** COVID-19, Zinc, Selenium, Nutritional status, Immunity

## Abstract

**Background:**

Without an adequate immune response, SARS-CoV2 virus can simply spread throughout the body of the host. Two of the well-known immunonutrients are selenium (Se) and zinc (Zn). Se and Zn deficiency might lead to inflammation, oxidative stress, and viral entry into the cells by decreasing ACE-2 expression; three factors that are proposed to be involved in COVID-19 pathogenesis. Thus, in the current study we aimed at evaluating the correlation between serum Se and Zn status and COVID-19 severity.

**Methods:**

Eighty-four COVID-19 patients were enrolled in this observational study. Patients were diagnosed based on an infectious disease specialist diagnosis, using WHO interim guidance and the recommendations of the Iranian National Committee of Covid-19. The patients with acute respiratory tract infection symptoms were checked for compatibility of chest computed tomography (CT) scan results with that of Covid-19 and Real-time polymerase chain reaction (RT-PCR) for corona virus infection. The severity of Covid-19 was categorized into three groups (mild, moderate, and severe) using CDC criteria. Serum Zn and Se level of all subjects was measured. The severity of the disease was determined only once at the onset of disease.

**Results:**

According to the results of linear regression test, there was a significant association between Zn and Se level and COVID-19 severity (β = − 0.28, P-value = 0.01 for Se; β = − 0.26, P-value = 0.02). However the significance disappeared after adjusting for confounding factors. Spearman correlation analysis showed a significant negative association between serum Zn, Se and CRP level (r = − 0.35, P-value = 0.001 for Se; r = − 0.41, P-value < 0.001 for Zn).

**Conclusion:**

Results suggest that increasing levels of Se and Zn were accompanied by a decrease in serum CRP level. However, the significant association between Se, Zn, and disease severity was lost after adjusting for confounding factors.

## Introduction

COVID-19 is a multi-organ disease that correlates with heightened intensive care support and a high morbidity rate [1]. Decreased immunity is a significant risk factor for viral respiratory infections. A proper diet and good nutritional status are important elements for an optimal immune response to prevent infection. Thus, a poor diet and nutritional deficiencies will increase the disease burden [2]. Two of the most important immunonutrients are selenium (Se) and zinc (Zn).

The biological effects of Se derive from 25 selenoproteins, the most well-known of which are selenoprotein P, iodothyronine deiodinase, thioredoxin reductase, and glutathione peroxidase (GpX). For example, a decrease in the GpX-1 level will lead to an increase in the production of reactive oxygen species (ROS), activation of NF-κB transcription, and an increase in oxidative stress and cell apoptosis [3]. Se is a well-known inhibitor of NF-kB and appears to decrease NF-kB-induced apoptosis and induce cytokine storms related to severe COVID-19 [4]. Recent studies have shown that the levels of selenoprotein P and GpX worldwide are borderline or below-optimal, even in many European countries [5]. A study in China found that the mean hair selenium level was high in a Chinese city with a high COVID-19 recovery rate and low in cities with high COVID-19 mortality rates [6]. Another population-based study and one cross-sectional study showed that Se deficiency correlated with the risk of COVID-19 mortality [7].

Up to 30% of common colds are caused by coronavirus infections [3]. Studies report that any decrease in the severity, duration and frequency of the common cold after Zn supplementation will depend on the Zn compound used, the dosage, and onset of use after symptom initiation [8]. In critically ill patients, persistently low serum Zn levels have an inverse correlation with recurrent sepsis and mortality from *Streptococcus pneumoniae* [9].

Infections with coronaviruses are accompanied by ciliary dyskinesia that result in impairment of mucociliary clearance. Physiological concentrations of Zn positively affect the length and number of bronchial cilia, as well as the ciliary beat frequency, which will improve the mucociliary clearance of viruses [8]. Ex-vivo studies showed that a low Zn level increased the permeability of the epithelium of the respiratory tract [10] and Zn supplementation enhanced lung integrity by inducing the expression of tight junction proteins (i.e. ZO-1 and claudin-1) [11]. Moreover, high Zn levels protect the lung from damage caused by mechanical ventilation [12]. Zn adequacy can decrease viral entry into the cells by decreasing ACE-2 expression, inhibiting fusion with the host membrane and suppressing the RNA-dependent RNA polymerase of the virus.

A low serum Zn level has been correlated with increased ROS and pro-inflammatory markers [8]. COVID-19-induced coagulopathy caused by microangiopathic organ failure, venous thromboembolism, and atherosclerosis development, which are the principal causes of death in these patients [13, 14]. Zn deficiency could promote thrombocyte aggregation and coagulation by inducing ROS production in platelets [15, 16]. Leukocytosis and neutrophilia have been associated with a poor prognosis for COVID-19. Recovery from lymphopenia can improve clinical recovery [17]. Zn supplementation could reverse lymphopenia in innate immune cells. Zn is also a necessary regulator of TLR-3 and TLR-4 induced signaling pathways [18].

The only previously published observational study on the relationship between Zn deficiency and COVID-19 output reported a near significant relationship between Zn deficiency and longer hospital stays, as well as a higher prevalence of respiratory distress syndrome []. Few studies have been published about the link between COVID-19 outcomes and serum levels of Se and Zn. To the best of our knowledge, no study has assessed the association between serum Zn and Se level and COVID-19 severity. The present study investigated the potential link between serum levels of Se and severity of COVID-19.

## Methods

This was an observational prospective study which evaluated 84 patients diagnosed with COVID-19 presenting to the emergency ward of Sina Hospital, which is affiliated with Tehran University of Medical Sciences (TUMS). The data were collected up to 1 September 2020. The study was approved by the ethics committee of the Tehran University of Medical Sciences (IR.TUMS.VCR.REC.1399.134).

Subjects were diagnosed by an infectious disease specialist using WHO interim guidance and the recommendations of the Iranian National Committee on COVID-19 [19]. Medical records were collected from the COVID-19 Registry (SHCo-19R) database of Sina Hospital [19]. SHCo-19R is a prospective, ongoing, hospital-based registry of patients diagnosed with COVID-19 who presented at the emergency ward of Sina Hospital.

The patients were 18 years of age and older with acute respiratory tract infection symptoms (e.g. cough, fever, dyspnea) with no other etiology that fully explained the clinical presentation. The compatibility of computed tomography (CT) chest scan results with that of COVID-19 detection and accurate diagnosis of a coronavirus infection from real-time polymerase chain reaction (RT-PCR) was used to detect the accuracy of diagnosis among patients. Evaluation of the severity of the disease was performed using CDC criteria for mild (fever, cough, sore throat, malaise, headache, muscle pain, nausea, vomiting, diarrhea, loss of taste and smell without shortness of breath, dyspnea, abnormal chest imaging), moderate (evidence of lower respiratory disease during clinical assessment or imaging, saturation of oxygen SpO_2_ ≥ 94% in room air at sea level at 5–6 days after infection), severe (SpO_2_ < 94% in room air at sea level, ratio of arterial partial pressure of oxygen to fraction of inspired oxygen (PaO_2_/FiO_2_) < 300 mm Hg, respiratory frequency > 30 breaths/min, or lung infiltrates > 50%. respiratory frequency within 24–48 h), and critical (septic shock, respiratory failure, and/or multiple organ dysfunction/failure).

Based on the available clinical data and the CDC criteria, we applied a severity risk score model that included three groups [20]. Patients manifesting mild and moderate symptoms were assigned to groups one and two, respectively. Patients with severe and critical symptoms were assigned to group three. Severity score at the time of admission was used for further evaluations. Abuse of alcohol or other substances, pregnancy or lactation, and renal and kidney failure were considered as exclusion criteria.

### Study measurements

The present study collected data on the demographic characteristics of age, gender, past medical history, baseline clinical characteristics of the disease, onset of symptoms, and comorbidities. The results of RT-PCR, radiology and laboratory were included.

### Serum Se, Zn and biochemical assessment

The serum level of Se and Zn was measured only at the onset of study. Fasting blood samples (5 ml) were collected from all subjects in the control and case groups at the time of admission and were centrifuged within 30 min of collection. The serum samples were kept at − 20 °C.

Serum Se and Zn were measured using flame atomic absorption spectrometry (FAAS) technique (Atomic absorption spectrophotometer AA-680; Shimadzu; Japan). Serum CRP levels were determined by the immunoturbidimetry method using a CRP reagent kit (Audit Diagnostics; Ireland). The ESR was detected using the Westergren method and D-dimer measurements were performed using a latex agglutination test (Biorex Fars; Iran). A human ferritin enzyme immunoassay test kit (Immunobiological Laboratories; Germany) was used to assess serum ferritin levels through enzyme-linked immunosorbent assay (ELISA). The troponin level was also determined using ELISA kits (Shanghai Crystal Day Biotech; China).

Complete blood counts and differential leukocyte counts were performed using a cell counter (Nihdon Kohden Celltac E; Japan). The prothrombin time (PT) and partial thromboplastin time (PTT) measurements were performed using the appropriate kits and the international normalized ratio (INR) was detected accordingly. The glucose level was done measured by the glucose oxidase method (intra 144 assay CV = 1.8%; Pars Azmoon; Iran). Serum creatinine levels were assessed using the photometric Jaffe method with commercially available kits (Pars Azmoon; Iran). The serum electrolyte levels for Ca, Na, and K were determined by the ion selective electrode principle. ALT and AST serum levels were measured using a laboratory test kit (Pars Lab; Iran). The level of LDH was also assessed using an LDH assay kit (Takara; Japan). The serum CPK activity was detected using an auto analyzer.

### Statistical analysis

Determination of sample size in this study was based on our recent experience with similar study design and no calculation for statistical power was conducted prior to the study onset. A Shapiro–wilk test was used to evaluate normality distribution of data. In normal distributed continuous data, one-way ANOVA was applied. In data which was not normally distributed, ANOVA test was performed on log transformed data. Games-Howell test was used for post-hoc analyses. The association between Se, Zn, and COVID-19 severity score was analyzed using linear regression for normally distributed data. Linear regression was performed on log transformed data for Skewed variables. The ANOVA and association results were also adjusted by confounding factors. The correlation between Se, Zn and CRP was assessed using Spearman correlation analysis. Values are stated as numbers, and percentages, means and standard deviations and median and interquartile range according to the type and the tests applied. P-value < 0.05 was considered as statistical significance level in all performed analysis. Analyses were carried out using SPSS 21. All tests were two-tailed.

## Results

### Baseline characteristics

Table 1 presents the baseline characteristics and clinical manifestations of COVID-19 among the study population according to the severity of the disease at base line. Overall, 38 patients (45.2%) experienced mild symptoms, 27 patients (32.1%) experienced moderate symptoms, and 19 patients (22.6%) experienced severe symptoms. Distribution of gender did not differ between groups of severity of COVID-19 (P-value = 0.372). The values were not significant for fever (p = 0.10), comorbid disease (p = 0.07), delusion p = 0.928), dizziness (p = 0.415), headache (p = 0.303), vertigo (p = 0.228), seizure (p = 0.356), myalgia (p = 0.109), respiratory symptoms (p = 0.771), lack of-appetite (p = 0.078), fatigue (p = 0.255), vomiting (p = 0.738), constipation (p = 0.555), diarrhea (p = 0.970), other gastrointestinal symptoms (p = 0.543), and dysphagia (p = 0.454). Table 1 shows that patients with severe COVID-19 were significantly older (81 ± 7 year) compared to patients in the mild (51 ± 14 year) and moderate (59 ± 14 year) groups (p < 0.001). Of these, 29.4% of patients were in the mild group, 37% in the moderate group, and 63.2% in the severe group required mechanical ventilation (p = 0.051).

**Table 1:** Baseline characteristics and clinical manifestations of the COVID-19 among studied participants according to the severity of disease

	COVID-19 disease severity			*P* value
	Mild n = 38 (45.2%)	Moderate n = 27 (32.1%)	Severe n = 19 (22.6%)	
Gender				
Number (%) of women	15 (39.5%)	15 (55.6%)	7 (36.8%)	
Number (%) of men	23 (60.5%)	12 (44.4%)	12 (63.2%)	0.372
Age (year)^a^ (mean, standard deviation)	51 ± 14	59 ± 14	81 ± 7	< 0.001
Having a history of chronic HTN, diabetes, CVDs or COPD	18 (50.0%)	19 (70.4%)	15 (78.9%)	0.07
Having history of neurological disease	10 (27.8%)	12 (44.4%)	6 (31.6%)	0.372
Delusion	1 (3.1%)	1 (3.7%)	1 (5.3%)	0.928
Dizziness	4 (12.5%)	5 (18.5%)	1 (5.3%)	0.415
Headache	7 (21.9%)	8 (29.6%)	2 (10.5%)	0.303
Vertigo	4 (12.5%)	4 (14.8%)	0 (0.0%)	0.228
Seizure	4 (12.5%)	1 (3.7%)	3 (15.8%)	0.356
Fever	20 (62.5%)	22 (81.5%)	10 (52.6%)	0.100
Mechanical ventilation	10 (29.4%)	10 (37.0%)	12 (63.2%)	0.051
Myalgia	11 (28.9%)	3 (11.1%)	3 (15.8%)	0.109
Respiratory symptoms	25 (65.8%)	17 (63%)	15 (78.9%)	0.771
Loss of appetite	9 (23.7%)	10 (37%)	2 (10.5%)	0.078
Fatigue	7 (18.4%)	9 (33.3%)	8 (42.1%)	0.255
Vomiting	8 (21.7%)	5 (18.5%)	3 (15.8%)	0.738
Constipation	1 (2.8%)	0 (0%)	1 (5.3%)	0.555
Diarrhea	3 (7.9%)	2 (7.4%)	2 (10.5%)	0.970
Other GI Symptoms^b^	5 (13.2%)	3 (11.1%)	1 (5.3%)	0.543
Dysphagia	0 (0%)	1 (3.7%)	1 (5.3%)	0.454

^a^Data are presented as mean (standard deviation)

^b^Other GI symptoms include: reflux, stomachache, and stomach tenderness

Additional file 1: Table summarizes the biochemical assessments of the COVID-19 patients according to severity. Serum level of CRP (P-value = 0.031), urea (P-value < 0.001), vitamin B12 (P-value = 0.001), Se (P-value = 0.01), INR (P-value < 0.001), and troponin (P-value = 0.007) were significantly different across severity group. Other biochemical parameters didn’t differ significantly among studied groups.

### Serum Zn and Se and COVID 19 severity

**Fig. 1:**
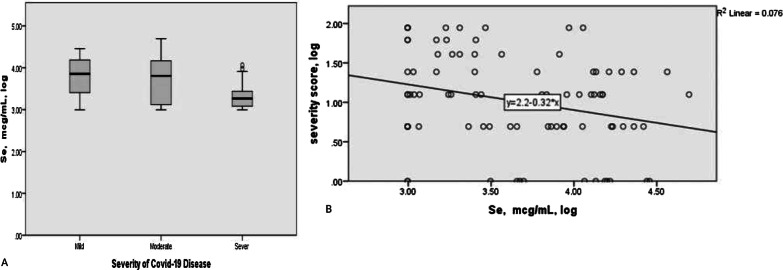
Comparison of log-transformed Se levels between COVID-19 cases according to disease severity categories. **A** the mean (SD) of log-transformed Se was 3.74 (0.48), 3.70 (0.56), 3.33 (0.33) in mild, moderate, and severe group respectively (p = 0.01). Serum Se level in patients with severe COVID­19 was significantly less than mild (p = 0.002) and moderate group (p = 0.02) (ANOVA test and Games-Howell post-hoc analyses). **B** scatter plot between log-transformed of Se and Severity score of covid-19 disease

**Fig. 2:**
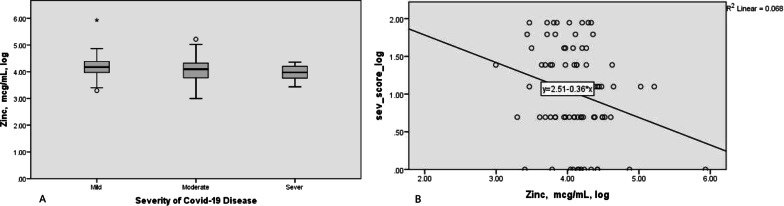
Comparison of log-transformed Zn levels between COVID-19 cases according to disease severity categories. **A** the mean (SD) of log-transformed Zn was 4.18 (0.44), 4.07 (0.46), 3.95 (0.29) in mild, moderate, and severe group respectively (p = 0.16) (ANOVA test). **B** scatter plot between log-transformed of Zn and Severity score of covid-19 disease

Figure 1 indicates serum Se levels between COVID-19 cases according to disease severity categories. Accordingly, the mean ± SD of serum Se were as follows: 47.07 ± 20.82 ng/ml, 47.36 ± 25.6 ng/ml, 29.86 ± 11.48 ng/ml in the mild, moderate and severe disease group respectively. According to simple linear regression model, there was a significant negative association between serum Se level and COVID-19 severity (standardized coefficient = − 0.28, P-value = 0.01). The association did not remain significant after adjusting for potential confounding factors including age and log-transformed of urea, CRP, INR, vitamin B12, lymphocyte count, hemoglobin, creatinine, troponin, and d-dimer (Table 2). Zn level was also significantly different across severity categories (standardized coefficient = − 0.26, P-value = 0.02). Similar to Se, after controlling the confounding effects (Inc. age and log-transformed of urea, CRP, INR, vitamin B12, lymphocyte count, hemoglobin, creatinine, troponin, and d-dimer) in multiple linear regression model, no significant association was observed between serum Zn level and COVID-19 severity. Comparison of log-transformed Se and Zn levels and COVID-19 severity was also presented in Figs. 1 and 2 using scatter plots.

**Table 2:** Un-standardized and standardized regression coefficients of log-transformed Zn and Se to log-transformed of covid-19 severity score in simple and multivariable * regression

	Independent variables	Unstandardized coefficient (95% CI)	SE	Standardized coefficient	P value
Simple linear	Zinc, mcg/ml, log	− 0.36 (− 0.66, − 0.07)	0.15	− 0.26	0.02
Regression	Se, mcg/ml, log	− 0.33 (− 0.57, − 0.08)	0.13	− 0.28	0.01
Multiple linear	Zinc, mcg/ml, log	− 0.12 (− 0.44, 0.21)	0.16	− 0.09	0.46
Regression	Se, mcg/ml, log	0.07 (− 0.2, 0.33)	0.13	0.07	0.62

*Analyses were controlled for the following covariates: age, and log-transformed of Urea, CRP, INR, VB 12, lymph, Hb, Cr, troponin, and d-dimer

### Serum concentrations of selenium, zinc and CRP in COVID-19 cases

**Fig. 3:**
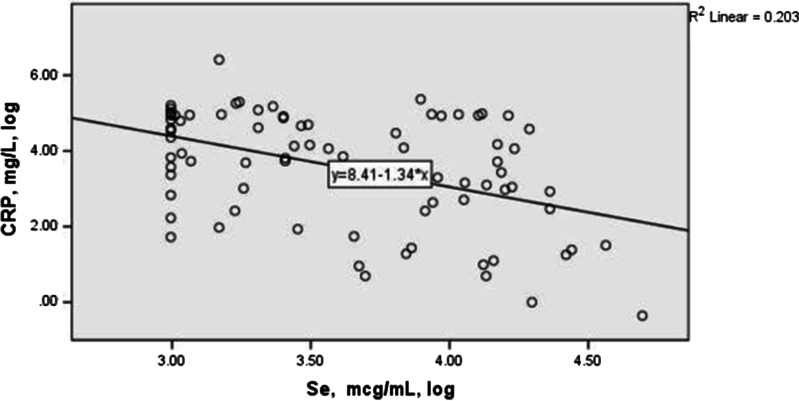
The correlation between log transformed of serum concentrations of selenium and CRP in the studied COVID-19 cases. The Spearman correlation between Se and PCR: − 0.41 (p < 0.001)

**Fig. 4:**
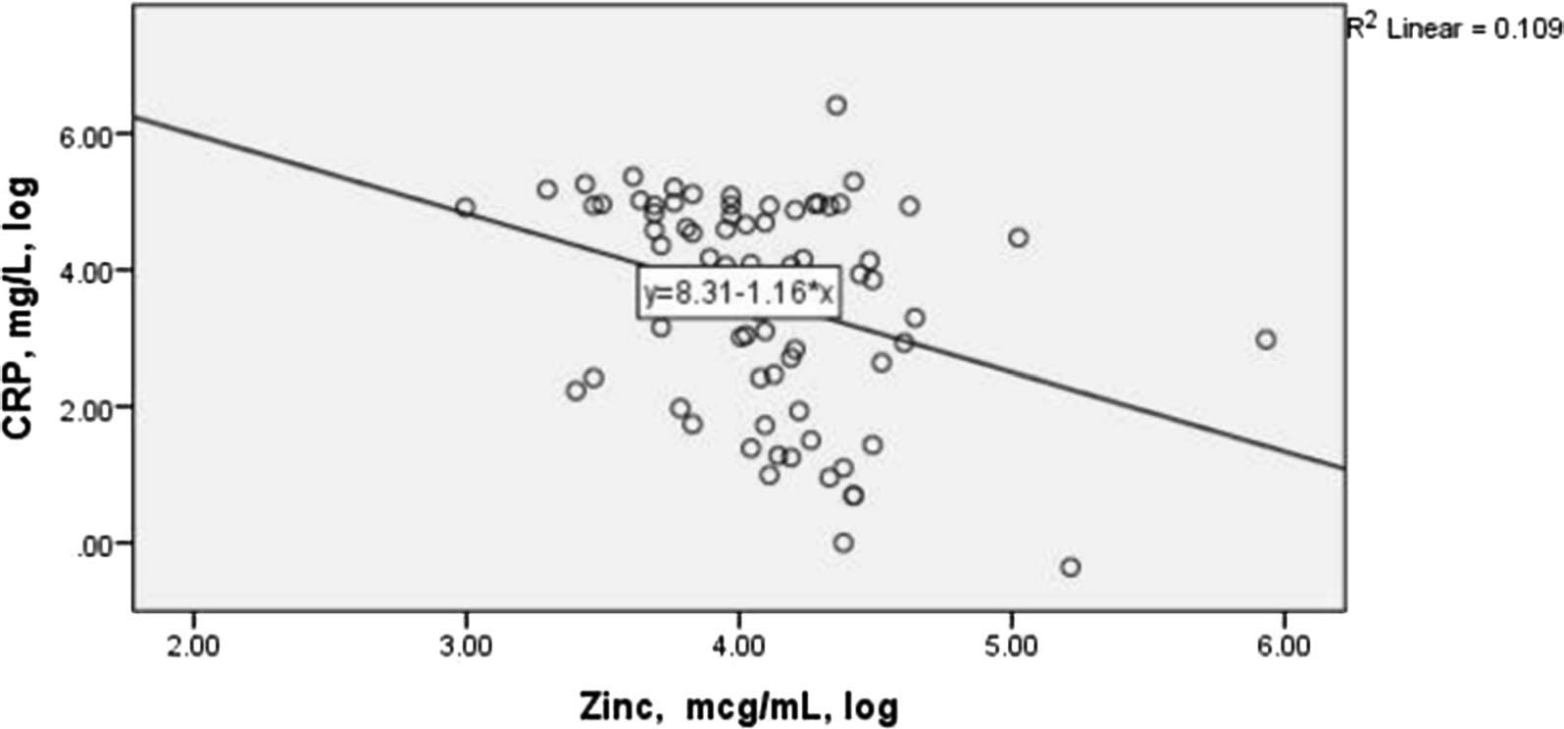
The correlation between log transformed of serum concentrations of zinc and CRP in the studied COVID-19 cases. The Spearman correlation between Zn and PCR was − 0.35 (p = 0.001)

Figures 3 and 4 describe the correlation between serum concentrations of selenium, zinc and CRP in the studied COVID-19 cases. Both minerals presented a statistically significant negative correlation with the inflammatory marker (CRP) levels using Spearman correlation analysis (r = − 0.35, P-value = 0.001 for Se; r = − 0.41, P-value < 0.001 for Zn).

## Discussion

To the best of our knowledge, this is the first study to investigate the association between Se and Zn status and COVID-19 severity [7]. In the current study, serum Se levels were significantly lower in patients with severe COVID-19 compared to patients with mild or moderate disease. A recent population-based study in 17 China cities have provided notable evidence about the relationship between cure rates from COVID-19 and Se status. They reported that, as the Se hair concentration in a population increased, the recovery rate from COVID-19 also increased [7].

The results of a clinical study in Germany showed a strong association between the serum Se level and COVID-19 outcomes in hospitalized patients, with 39% of survivors and 65% of the deceased having low serum Se levels [8]. The results of another study were in agreement with our results by showing that, in a clinical trial, supplementation with selenium, magnesium and vitamin B12 reduced the need for oxygen and/or intensive care support [21]. However, after adjusting for confounding factors including age, urea, CRP, INR, vitamin B12, lymphocyte count, hemoglobin, creatinine, troponin, and d-dimer, the significance disappeared. It can be concluded that the effect of confounding factors especially age are stronger than the effect of serum selenium level in predicting COVID-19 severity. More ever, in current study Se level was far below the normal range (70–150 ng/ml) across three severity group (47.07 ± 20.82 ng/ml, 47.36 ± 25.6 ng/ml, 29.86 ± 11.48 ng/ml in the mild, moderate and severe disease group respectively) [22]. Se sufficiency is needed for observing its anti-inflammatory and immune-enhancing effect in COVID-19 patients.

We observed a significant relationship between Se and CRP level. In accordance with our results, preclinical and clinical studies of other inflammatory diseases have shown a relationship between serum CRP and Se level. In a study on 137 critically ill children, an inverse association was observed between CRP and serum Se level [23]. A preclinical study on mice assessed the oxidative stress and expression of inflammatory markers of primary-cultured peritoneal macrophages in Se-deficient and control groups [23]. Se deficiency was found to ameliorate the antioxidant capacity and triggered the accumulation of oxygen free radicals. Se deficiency also significantly promoted the expression of the inflammatory mediators of IL-12, iNOS, IL-1β, NF-κB, and PTGe. An increase in NF-κB expression was followed by the accumulation of oxygen free radicals, which hindered the phagocytic capacity of the macrophages [24].

GpX and thioredoxin reductase play a pivotal role in modulating the inflammatory response and mediating the regulation of T-cell activity. Se prevents oxidative damage to endothelial cells and preserves their function. One life-threatening feature of COVID-19 is its involvement in thrombotic events such as deep-vein and arterial thrombosis, large vessel clots, microvascular thrombosis, and pulmonary embolism, probably caused by endothelium dysfunction, platelet activation, and inflammation.

SARS-CoV-2 has been shown to enhance endotheliitis induced by the infection of endothelial cells and the inflammatory response of the host. COVID-19 also causes thrombocytopenia and can induce stroke, even in young patients. The formation of thromboxane A2 (TxA2) is a key element in platelet aggregation and activation that can lead to blood thrombosis/coagulation. Sodium selenite has been reported to have antiaggregating properties from the reduction of TxA2 formation [1].

COVID-19 is more prevalent and more severe in older patients [25]. Low or marginal Se levels are more prevalent and have been reported to be associated with increased ICU admission rates [26]. In Sweden, 71% of older adults admitted to the ICU were Se deficient [27]. Supplementation of Se for the institutionalized elderly was found to reduce the infection rate significantly [26].

In current study, we observed a significant association between Zn level and COVID-19 severity. Similar to Se, the association did not remain significant following the adjustment of confounding factors. We observed a significant negative relationship between serum Zn and CRP level. A number of review articles have examined the relationship between Zn level and COVID-19 [8, 28, 29] and discussed the relationship between Zn deficiency and deep respiratory infections other than COVID-19. Their findings concluded that Zn deficiency could be linked to the risk of infection and severe complication of COVID-19 [8, 28, 29].

The only study available about Zn levels in Covid-19 patients showed that it was significantly lower in COVID-19 patients than the healthy control subjects. Deficient patients had higher rates of hospital stay, acute respiratory distress syndrome, and mortality []. Finding no association between Zn level and disease severity, could be explained by the stronger effect of confounding factors. Also it could in part be explained by the very low Zn serum levels in studied subjects. Serum Zn level in the majority of studied population was below 70 mcg/ml that considered as the minimum of the normal serum range [30]. Zn adequacy is required to provide its immune-enhancing, anti-inflammatory, and other protective effects in COVID-19 patients. Available evidence also suggests that Zn deficiency is highly prevalent in Iran secondary to high phytate intake [31].

Because of the low proportion of patients with mortality in the current study we failed to assess the correlation between the mortality from disease and serum levels of Zn and Se. Furthermore, due to the observational structure of the current study, we couldn’t describe the cause and effect relationship between serum Zn and Se level and COVID-19. Hence, further prospective cohort studies are needed. Another limitation of the present study was that we failed to follow up and assess patients in different stages of disease as we only collected the serum Se and Zn levels once at the onset of admission to hospital. However, it is recommended to be considered for further research in this field of study. According to the existing methodological issue, the current study results should be considered cautiously. Moreover, further studies are need to explore whether Zn and Se supplementation can improve COVID-19 characteristics and if so, unfold the undiscovered cellular or molecular mechanisms regarding the alleviating impact of this minerals on COVID-19 symptoms.

## Conclusion

Taken together, these results proposed that higher level of serum Se and Zn was accompanied by reduction of CRP level. In addition to age and inflammatory biochemical parameters, to a lesser extent, Zn and Se level could take part in predicting COVID-19 severity. Although the results of the current study is promising, the hypothesis still need to be tested by thoroughly monitored interventional studies under high quality standards to enlighten the potential role of Se and Zn in COVID19 disease and unfold probable adverse relation between this mineral’s level and COVID-19.

## Supplementary Information

**Additional file 1: Table.** Biochemical assessments of the COVID-19 patients according to the severity of COVID-19.

## Data Availability

The datasets used and/or analyzed during the current study are available from the corresponding author on reasonable request.
