# Supplementary material for: The correlation between serum selenium, zinc, and COVID-19 severity: an observational study
Source: BMC Infect Dis. 2021 Sep 3;21:899. doi: 10.1186/s12879-021-06617-3 (PMC8414458; doi:10.1186/s12879-021-06617-3)
Supplement: Supplementary file 1 — Additional file 1: Table. Biochemical assessments of the COVID-19 patients according to the severity of COVID-19. [file 12879_2021_6617_MOESM1_ESM.docx]

**Additional Table**: **Biochemical assessments of the COVID-19 patients according to the severity of COVID-19.**

|  | **COVID-19 disease severity** | | |  |
| --- | --- | --- | --- | --- |
|  |  | | |  |
|  | **Mild**  **n=38**  **(45.2%)** | **Moderate**  **n= 27**  **(32.1%)** | **Severe**  **n=19**  **(22.6%)** |  |
|  | **Median (Interquartile range)** | **Median**  **(Interquartile range)** | **Median**  **(Interquartile range)** | **P value** |
| **O2saturation %** | 94.00 (6.75) | 92.00 (17) | 91.00 (7) | 0.159 |
| **Log-transformed** | 4.52 (0.08) | 4.44 (0.2) | 4.47 (0.14) | 0.12 |
| **Onset to admission days** | 6 (6) | 7 (10) | 7 (27) |  |
| **Log-transformed** | 1.71 | 1.98 | 2.10 | 0.378 |
| **Urea mg/dl** | 26.00 (19.5) ^a^ | 30.00 (22) ^b^ | 61.00 (78) ^a,b^ | 0.000 |
| **Log-transformed** | 33.96 ( 0.50 ) | 37.81 (0.58) | 76.73 (0.69 ) | 0.00 |
| **CRP, mg/L** | 24.0 (89.8) ^a^ | 62.2 (118.7) | 100.9 (105) ^a^ | 0.023 |
| **Log-transformed** | 3.04 (1.54) | 3.69 (1.54) | 4.28 (1.05) | 0.012 |
| **INR** | 1.04 (0.1) ^a^ | 1.13 (0.24) | 1.35 (0.35) ^a^ | 0.000 |
| **Log-transformed** | 0.64 ( 0.12) | 0.15 (0.17) | 0.34 (0.29) | 0.00 |
| **White blood cell count, × 109/L** | 8.55 (7.74) | 8.90 (11.40) | 8.70 (8.20) | 0.721 |
| **Log-transformed** | 2.21 (0.90) | 2.33 (0.91) | 2.14 (0.66) | 0.74 |
| **Lymphocyte count × 109/L (%)** | 17.25 (18.83) | 18.00 (15.90) | 13.70 (13.30) | 0.132 |
| **Log-transformed** | 2.72 (0.76) | 2.79 (075) | 2.31 (0.78) | 0.09 |
| **Hemoglobin, g/L** | 14.20 (3.40) | 12.80 (3.50) | 11.50 (5.20) | 0.136 |
| **Log-transformed** | 2.55 (0.17) | 2.44 (0.35) | 2.61 (0.58) | 0.18 |
| **Platelet count, ×109/L** | 201.50 (99.25) | 204.00 (156) | 221.00 (200) | 0.409 |
| **Log-transformed** | 4.87 (1.2) | 5.29 ( 0.76) | 5.24 (0.72) | 0.21 |
| **Plasma glucose,**  **mg/dl** | 104.00 (55) | 124.00 (61) | 111.00 (38) | 0.809 |
| **Log-transformed** | 4.35 (0.20) | 4.27 (0.16) | 4.30 (0.10) | 0.11 |
| **Serum sodium, meq/dl** | 136.00 (7.25) | 134.00 (6) | 134.00 (7) | 0.243 |
| **Log-transformed** | 4. 97 (0.39) | 4.90 (0.31) | 4.89 (0.07) | 0.40 |
| **Serum calcium, mg/dl** | 8.50 (0.83) | 8.30 (1.30) | 8.25 (1.33) | 0.108 |
| **Log-transformed** | 2.07 (0.41) | 1.98 (0.49) | 2.07 (0.93) | 0.65 |
| **Serum potassium,**  **meq/dl** | 4.07 (0.7) | 4.40 (0.9) | 4.23 (1.49) | 0.310 |
| **Log-transformed** | 1.41 (0.15) | 1.46 (0.18) | 1.50 (0.19) | 0.22 |
| **Creatinine,**  **mg/dl** | 1.00 (0.45) | 1.00 (0.87) | 1.30 (1.78) | 0.271 |
| **Log-transformed** | 0.04 (0.41) | 0.22 (0.63) | 0.36 (0.65) | 0.11 |
| **PT, s** | 14.20 (2.05) | 16.00 (5.10) | 16.40 (5.20) | 0.075 |
| **Log-transformed** | 2.77 (0.49) | 2.88 (0.83) | 2.83 (0.31) | 0.78 |
| **PTT, s** | 37.60 (9.55) | 39.75 (19.68) | 37.60 (22.30) | 0.840 |
| **Log-transformed** | 3.62 (0.28) | 3.66 (0.43) | 3.67 (0.45) | 0.87 |
| **AST, U/L** | 43.00 (31.25) | 42.00 (25) | 46.50 (30.25) | 0.983 |
| **Log-transformed** | 3.87 (0.72) | 3.81 (0.49) | 3.88 (0.49) | 0.91 |
| **ALT, U/L** | 36.50 (18) | 36.00 (22) | 29.50 (27) | 0.547 |
| **Log-transformed** | 3.64 (0.52) | 3.59 (0.54) | 3.60 (0.70) | 0.93 |
| **ESR, mm** | 36.00 (58) | 64.50 (59.75) | 55.00 (67) | 0.286 |
| **Log-transformed** | 3.44 (1.02) | 3.77 (0.97) | 3.81 (0.83) | 0.31 |
| **LDH, U/L** | 550.50 (195) | 524.00 (330) | 532.50 (198.75) | 0.750 |
| **Log-transformed** | 6.33 (0.36) | 6.25 (0.40) | 6.18 (0.58) | 0.51 |
| **CPK, U/L** | 149.00 (284.5) | 127.50 (126) | 78.00 (201.5) | 0.398 |
| **Log-transformed** | 5.14 (1.15) | 4.82 (1.54) | 4.31 (2.01) | 0.262 |
| **D_Dimer ug/mL** | 663.00 (1106.50) ^a^ | 1034.14 (630.37) | 1970.00 (1886) ^a^ | 0.046 |
| **Log-transformed** | 6.48 (1.12) | 6.91 (0.89) | 7.08 (1.56) | 0.29 |
| **Ferritin ng/mL** | 245.70 (387.75) | 232.23 (477.80) | 408.00 (1465.63) | 0.541 |
| **Log-transformed** | 5.68 (0.83) | 5.55 (0.84) | 6.04 (1.11) | 0.33 |
| **Troponin ng/mL** | 3.60 (7.70) ^a^ | 9.55 (43.5) | 23.00 (79.5) ^a^ | 0.002 |
| **Log-transformed** | 1.61 (1.49) | 2.43 (1.87) | 3.27 (1.71) | 0.00 |
| **Zinc,**  **mcg/mL** | 65.00 (28) | 60.00 (37) | 53.00 (26) | 0.137 |
| **Log-transformed** | 4.18 (0.44) | 4.07 (0.46) | 3.95 (0.29) | 0.15 |
| *CRP* C-reactive Protein, *INR* International Normalized Ratio, *ESR* Erythrocyte Sedimentation rate, *PT* Prothrombin Time, *PTT* Partial Thromboplastin Time, *ALT* Alanine transaminase, *AST* aspartate aminotransferase, *LDH* lactate dehydrogenase, *CPK* Creatine phosphokinase;  **p-value from Kruskal Wallis test for raw data and ANOVA test for log-transformed data** | | | | |
